# Effects of *Plotytarya strohilacea Sieb. et Zuce* Tannin on the Growth Performance, Oxidation Resistance, Intestinal Morphology and Cecal Microbial Composition of Broilers

**DOI:** 10.3389/fvets.2021.806105

**Published:** 2022-01-05

**Authors:** Zhenkai Tong, Fuhong Lei, Lixuan Liu, Fei Wang, Aiwei Guo

**Affiliations:** ^1^Faculty of Life Sciences, Southwest Forestry University, Kunming, China; ^2^Moringa oleifera Research Center, Yunnan Institute of Tropical Crops, Jinghong, China

**Keywords:** *Plotytarya strohilacea Sieb. et Zuce* tannin, growth performance, intestinal morphology, cecal microbial, broilers

## Abstract

The purpose of this experiment was to study the effects of *Plotytarya strohilacea Sieb. et Zuce* tannin on broilers growth performance, antioxidant function, intestinal development, intestinal morphology and the cecal microbial composition. In this experiment, a total of 360 1-day-old Arbor Acres male broilers were randomly divided into 4 treatment groups, with 6 replicates in each group and 15 broilers in each replicate. The control group (Control) was fed the basal diet, and the broilers were fed a basal diet supplemented with 0 (Control), 100 (PT1), 400 (PT2), and 800 (PT3) mg/kg *Plotytarya strohilacea Sieb. et Zuce* tannins for 42 days, respectively. The results showed that the average daily feed intake (ADFI) of the PT1 group was significantly lower than that of the control group, and there was a significant quadratic relationship between the ADFI and the concentration of tannin (*P* < 0.05). Compared with the control group, the F/G of broilers during the 22–42 days phase in the PT1 group showed a decreasing trend (*P* = 0.063). The serum catalase (CAT) activity in the PT1 group was significantly higher than those of the other three groups, and the effect was significantly quadratically related to the dosage (*P* < 0.05). The glutathione peroxidase (GSH-Px) activity in the PT1, PT2 and control groups were significantly higher than that of the PT3 group, and the effect was significantly quadratically related to the addition amount (*P* < 0.05). The serum total antioxidant capacity (T-AOC) activity in the PT1 group was significantly higher than that in the control group, and the effect was significantly quadratically related to the addition amount (*P* < 0.05). Compared to the control group, the villus height of jejunum in the PT1, PT2 and PT3 groups were significantly higher, and there was a significant quadratic relationship between the villus height of jejunum and the addition amount (*P* < 0.05). In addition, adding tannins to diets significantly increased *Parabacteroides* in the dominant genus (*P* < 0.05). In conclusion, dietary supplementation with *Plotytarya strohilacea Sieb. et Zuce* tannin improved the growth performance, antioxidant function, and intestinal morphology along with an increased abundance of *Parabacteroides* in the cecum, and the recommended dosage of tannin in broiler diets was 100 mg/kg.

## Introduction

With the rapid development of livestock and poultry, long-term and low-dose preventive drugs or excessive use and abuse of antibiotics and other factors result in the presence of antibiotic residues in livestock and poultry products, the resistance of pathogens to antibiotics, the imbalance of normal microbial flora, the decline of animal immunity, and related effects (1–3). With the increasing food safety awareness of the population and the implementation of the policy of antibiotic prohibition in various countries worldwide to control the use of antibiotics, the cost of poultry production will increase (4). The search for antibiotic alternatives with natural, inexpensive, non-toxic and residual characteristics has become a study focal point in the poultry industry (5).

*Platycarya strobilacea Sieb. Et Zucc*, a walnut plant, is widely distributed in northwest China, east China, central China, south China and other places. *Platycarya strobilacea Sieb. Et Zucc* is widely used in traditional medicine in China with a long history. It can dispel wind, dispel phlegm, relieve dryness and kill insects (6, 7). Allspice fruit extract is derived from the fruit sequence of *Platycarya strobilacea Sieb. Et Zucc* and is a natural product that includes plant polyphenols, flavonoids, ellagic acid, vitamins, tannins and other bioactive substances (8, 9). The tannins are ellagic tannins, and the main ingredient is oak ellagin equine, which can be obtained by the hydrolysis of ester ellagic tannins formed by two hexahydroxybiphenyl diacyl groups and glucose. Tannins have special biological and pharmacological activities, and previous studies have mainly focused on the inhibition of cell toxicity, harmful intestinal bacteria, and oxidation and the prevention of cancer and anti-aging effects (10, 11). Tannins have also been used as a compound Chinese medicine in the treatment of acute chronic rhinitis and sinusitis (12). Faraha et al. (13) have studied that grape seed extracts which contain tannins significantly decreased serum total cholesterol, low-density lipoprotein cholesterol and meat malondialdehyde level, and increased the antibody titer against Newcastle disease virus vaccine of broilers. However, few studies have compared and analyzed the effects of *Platycarya strobilacea Sieb. Et Zucc* tannins on the growth performance, intestinal development and intestinal microbial community of poultry based on microbial sequencing technology.

In this study, *Platycarya strobilacea Sieb. Et Zucc* tannins were used as an antibiotic substitution to determine their effects on the growth performance of broilers. Additionally, 16S rDNA sequencing was used to compare the effects of tannins on the intestinal microbial community of these broilers, in combination with intestinal development and intestinal morphology, to better understand the impact of tannins on the growth performance of broilers.

## Methods

### Experimental Design and Animal Management

In this experiment, a total of 360 1-day-old Arbor Acres male broilers were randomly divided into 4 treatment groups, with 6 replicates in each group and 15 broilers in each replicate. The broilers were fed a basal diet supplemented with 0 (Control), 100 (PT1), 400 (PT2), and 800 (PT3) mg/kg *Platycarya strobilacea Sieb. Et Zucc* tannins, respectively. The tannins with a content of ≥ 65% tannins were provided by the Research Group on the Chemical Utilization of Plant Tannins, Institute of Chemical Industry of Forest Products, CAF. The testing period was from 1 to 42 days of age. Two feeding phase diets were utilized: a starter diet from 1 to 21 days and a grower diet from 22 to 42 days (Table 1). The diets were formulated to meet the nutrient requirements recommended by the National Research Council (14). Birds had *ad libitum* access to feed and water and were reared with 23 h of light per day. Animal use and care were approved by the Academic Committee of Southwest Forestry University. Every effort was made to reduce animal stress. Daily, health status was observed and dead chickens and feed consumption were recorded.

**Table 1:** Composition and nutrient levels of basal diets (air-dry basis).

**Ingredients**	**Content (%)**		**Nutrient level**	**Content (%)**	
	**1–21 days**	**22–42 days**		**1–21 days**	**22–42 days**
Corn	56.68	59.22	ME (MJ/kg)^*c*^	12.30	13.05
Soybean oil	2.00	3.50	CP	21.00	19.00
Soybean meal (43%)	36.80	33.24	*D*-Lys	1.20	1.00
Lysine hydrochloride (98%)	0.30	0.20	*D*-Met+Cys	0.85	0.80
*DL*-Met	0.30	0.27	*D*-Thr	0.65	0.60
CaCO_3_	1.10	1.05	*D*-Trp	0.23	0.20
CaHPO_4_	2.20	1.90	Ca	1.00	0.90
NaCl	0.30	0.30	Total P	0.68	0.55
Choline chloride (70%)	0.09	0.09	NPP	0.45	0.35
Vitamin premix ^a^	0.03	0.03			
Mineral premix ^b^	0.20	0.20			
Total	100	100			

*^a^The vitamin premix provided for each kilogram of diet: Vitamin A, 8000 IU; Vitamin B_1_, 2 mg; Vitamin B_2_, 9.60 mg; Niacin, 35 mg; Vitamin B_6_, 3.5 mg; Biotin, 0.18 mg; Folic Acid, 0.55 mg; Vitamin B_12_, 0.01 mg; Vitamin D_3_, 1000 IU; Vitamin E, 20 IU; Vitamin K_3_, 0.5 mg; Calcium Pantothenate, 10 mg.*

*^b^The mineral element premix provided for each kilogram of diet: Fe 80 mg; Zn 80 mg; Mn 100 mg; I 0.7 mg; Cu 8 mg; Se 0.3 mg.*

*^c^ME was calculated value, while the others were measured values.*

*NPP, non-phytate phosphorus*.

### Growth Performance

The feed intake and body weight of birds were recorded on days 21 and 42. The average daily feed intake (ADFI), average daily gain (ADG), and feed:gain ratio (F/G) were calculated for days 1–21, days 22–42 and days 1–42.

### Sample Collection

On day 42, 6 birds per treatment group with similar weight per group were selected from 6 replicates and euthanized by severing the jugular vein after blood sampling collection from the wing vein with vacuum blood collection tubes. Approximately 2 ml of blood in coagulation-promoting tubes was collected and centrifuged at 4,000 × g for 10 min to obtain serum and was then stored at −80°C until analysis. The lengths of the duodenum and cecum (both sides) were measured, and the corrected lengths of the duodenum and cecum were calculated according to the formula: Duodenal/Cecum length (cm/kg) = Duodenal/Cecum length (cm)/live weight (kg). Then, cecal chyme was collected, immersed in liquid nitrogen, and stored at −80°C for DNA extraction and 16S rDNA amplicon sequencing analysis by Novogene Corporation (Beijing, China). Then, the weights of the duodenum and cecum (both sides) were measured (contents removed), and the duodenum and cecum indices were calculated according to the formula: Duodenal/Cecum index (%) = [Duodenal/Cecum weight (g)/live weight (kg)] ^*^ 100. Approximately 2 cm of intestinal tissue from the jejunum was excised, emptied of chyme, and then fixed with 4% paraformaldehyde solution.

### Serum Antioxidant Indices

Serum was used to test blood antioxidant indices. According to the kit instructions, the total superoxide dismutase (T-SOD), catalase (CAT), glutathione peroxidase (GSH-Px), total antioxidant capacity (T-AOC) and malondialdehyde (MDA) activities in the serum and liver were detected by colorimetry. All commercial antioxidant indicator kits were purchased from Nanjing Jiancheng Bioengineering Institute.

### Intestinal Morphology

Intestinal segments (2 cm) in the middle of jejunum and ileum were taken and stained with hematoxylin and eosin (H&E). Consecutive sections of jejunum and ileum (5 m) were prepared for histomorphological observation. The well-oriented villi and the associated crypt of each sample were selected for morphological analysis using Leica Microsystems. The villus height and crypt depth were measured. Then the ratio of villus height to crypt depth (V/C) was then calculated.

### DNA Extraction and Sequencing Library Construction of Cecal Chyme

A total of 500 ± 50 mg of cecal chyme was randomly weighed from each replicate using a DNA Kit (DP328, Tiangen Biotechnology Co., Ltd.) to extract the total genomic DNA, and the integrity and concentration of RNA were detected by a NanoDrop ND 2000 (Thermo, America). According to the target fragment, PCR amplification of the V4 region of 16S rDNA and 1.5% agarose gel electrophoresis were performed to extract 400–450 bp PCR products, which were purified using a GeneJET Gel Extraction Kit (Thermo, America). The library was constructed with an Ion Plus Fragment Library Kit (Thermo Fisher Scientific, Waltham, MA, USA). After Qubit quantification and library testing, the constructed library was sequenced using the IonS5TMXL sequencing platform at Novogene Bioinformatics Technology Co., Ltd. (Beijing, China).

### Quality Filtering and Sequence Data Analysis

Based on the IonS5^TM^ XL sequencing platform, the raw tag quality was filtered by FLASH (V1.2.7), and effective tags were obtained. All effective tags were clustered into operational taxonomic units (OTUs) with 97% identity, and then the OTU sequences and Silva132 database were used for species annotation analysis. The dominant species (relative abundance > 2%) at the family and genus levels were analyzed according to species annotation results. For alpha diversity measurements, the alpha diversity indices were calculated based on the OTUs using the Shannon, Simpson and Chao1 indices. LEfSe analysis was employed to identify the biological differences between treatments.

### Statistical Analysis

All data are presented as means with pooled SEM values. Statistical analyses were carried out with SPSS 22.0 for Windows (SPSS Inc., Chicago, IL, United States). One-way ANOVA followed by the LSD multiple comparison test was used to evaluate the differences among the treatment groups. *P* ≤ 0.05 was considered statistically significant. Probabilities of *P* > 0.05 but *P* < 0.10 were defined as tendencies.

## Results

### Growth Performance

Table 2 shows the effects of *Plotytarya strohilacea Sieb. et Zuce* tannin on the broilers' growth performance. The ADFI of broilers during the days 1–21 phase in the PT1 group was significantly lower than that in the control group, and there was a significant quadratic correlation between ADFI and the amount of tannin (*P* < 0.05). Compared with the control group, the F/G of broilers during the days 22–42 phase in the PT1 group showed a decreasing trend (*P* = 0.063).

**Table 2:** Effects of *Plotytarya strohilacea Sieb. et Zuce* tannin on broilers growth performance.

**Days of age**	**Items**	**Groups**				**SEM**	***P*** **-value**		
		**Control**	**PT1**	**PT2**	**PT3**		**ANOVA**	**Linear**	**Quadratic**
1–21 days	ADG (g)	50.30	48.73	49.86	50.72	0.383	0.282	0.82	0.062
	ADFI (g)	66.50^a^	61.43^b^	64.16^ab^	63.91^ab^	0.574	0.015	0.078	0.015
	F/G	1.32	1.26	1.29	1.26	0.006	0.221	0.662	0.852
22–42 days	ADG (g)	119.69	118.20	116.04	118.23	1.085	0.711	0.41	0.787
	ADFI (g)	195.92	188.14	190.87	195.35	1.820	0.373	0.511	0.107
	F/G	1.64	1.59	1.64	1.65	0.009	0.063	0.89	0.042
1–42 days	ADG (g)	85.38	83.46	82.95	83.72	0.645	0.576	0.212	0.656
	ADFI (g)	130.55	124.78	127.51	128.91	1.067	0.260	0.404	0.089
	F/G	1.53	1.50	1.54	1.54	0.006	0.102	0.898	0.048

*In the same row, values with no letter or the same letter superscripts mean no significant difference (P > 0.05), while with different small letter superscripts mean significant difference (P < 0.05). The same as below.*

*ADG, average daily gain; ADFI, average daily feed intake; F/G, feed:gain ratio*.

### Antioxidant Activity

Table 3 shows the effects of *Plotytarya strohilacea Sieb. et Zuce* tannin on antioxidant activity of broilers at 42 days of age. The serum CAT activity of broilers in the PT1 group was significantly higher than that of control, PT2 and PT3 groups, and there was a significant quadratic correlation between CAT activity and the amount of tannin (*P* < 0.05). The GSH-Px activity of broilers in the PT1, PT2 and control groups were significantly higher than that in the PT3 group. The activity of GSH-Px was significantly correlated with the addition amount of tannin (*P* < 0.05). The serum total antioxidant capacity (T-AOC) of broilers in the PT1 group was significantly higher than that in the control group. There were significant linear and quadratic correlations between T-AOC capacity and the amount of tannin (*P* < 0.05).

**Table 3:** Effects of *Plotytarya strohilacea Sieb. et Zuce* tannin on serum antioxidant activity of broilers at 42 days of age.

**Items**	**Groups**				**SEM**	***P*** **-value**		
	**Control**	**PT1**	**PT2**	**PT3**		**ANOVA**	**Linear**	**Quadratic**
CAT (U/ml)	11.55^b^	26.07^a^	13.94^b^	9.77^b^	1.613	0.001	0.357	0.001
T-SOD (U/ml)	105.99	113.14	112.01	105.64	2.019	0.432	0.576	0.136
GSH-Px (U/ml)	967.57^a^	1105.87^a^	1091.11^a^	645.51^b^	60.793	0.028	0.442	0.005
T-AOC (U/ml)	0.30^b^	0.63^a^	0.45^ab^	0.44^ab^	0.038	0.011	0.018	0.018
MDA (nmol/ml)	1.91	2.10	1.81	1.82	0.178	0.932	0.912	0.618

*CAT, catalase; T-SOD, total superoxide dismutase; GSH-Px, glutathione peroxidase; T-AOC, total antioxidant capacity; MDA, malondialdehyde; Means with different superscript letters within a row are significantly different (P < 0.05)*.

### Length and Weight of Intestines

Table 4 shows the effects of *Plotytarya strohilacea Sieb. et Zuce* tannin on the correction length and index of broilers intestines at 42 days of age. There were no significant differences (*P* > 0.05) among any of the treatments for the corrected length and weight of broiler intestines at 42 days of age.

**Table 4:** Effects of *Plotytarya strohilacea Sieb. et Zuce* tannin on correction length and index of broilers intestines at 42 days of age.

**Items**	**Groups**				**SEM**	***P*** **-value**		
	**Control**	**PT1**	**PT2**	**PT3**		**ANOVA**	**Linear**	**Quadratic**
Duodenal length^a^ (cm/kg)	9.62	9.97	10.14	9.34	0.192	0.468	0.876	0.212
Cecum length^a^ (cm/kg)	6.34	6.83	6.71	6.25	0.103	0.133	0.508	0.028
Duodenal index^b^ (%)	0.39	0.39	0.41	0.37	0.013	0.709	0.848	0.453
Cecum index^b^ (%)	0.27	0.30	0.24	0.27	0.008	0.097	0.873	0.188

*^a^Duodenal/cecum length (cm/kg), Duodenal/cecum length (cm)/live weight (kg).*

^b^Duodenal/cecum index (%), [Duodenal/cecum weight (g)/live weight (kg)] ^*^ 100.

### Intestinal Morphology

Table 5 shows the effects of *Plotytarya strohilacea Sieb. et Zuce* tannin on intestinal morphology of the broilers at 42 days of age. The villus heights of the jejunum in the PT1, PT2 and PT3 groups were significantly higher than that in the control group, and there were significant linear and quadratic correlations between the villus height of the jejunum and the amount of tannin (*P* < 0.05). The V/C of the ileum in the control and PT1 groups were significantly higher than that in the PT2 group. There was a significant linear correlation between V/C and the amount of tannin (*P* < 0.05).

**Table 5:** Effects of *Plotytarya strohilacea Sieb. et Zuce* tannin on intestinal morphology of the broilers at 42 days of age.

**Items**	**Groups**				**SEM**	***P*** **-value**		
	**Control**	**PT1**	**PT2**	**PT3**		**ANOVA**	**Linear**	**Quadratic**
**Jejunum**								
Villus height/μm	1,254.58^c^	1,424.64^b^	1,780.80^a^	1,399.42^b^	48.191	0.001	0.002	0.001
Crypt depth/μm	185.74	199.41	205.16	214.53	9.160	0.725	0.288	0.728
V/C	6.75	7.14	8.68	6.52	5.261	0.356	0.338	0.373
**Ileum**								
Villus height/μm	1,149.86	1,279.52	1,203.08	1,343.76	45.470	0.436	0.203	0.672
Crypt depth/μm	137.22	156.84	221.34	208.19	16.763	0.212	0.078	0.567
V/C	8.38^a^	8.16^a^	5.44^b^	6.45^ab^	2.713	0.022	0.014	0.765

*V/C, the ratio of villus height to crypt depth. Means with different superscript letters within a row are significantly different (P < 0.05)*.

### Cecal Microbiota Composition

**Figure 1:**
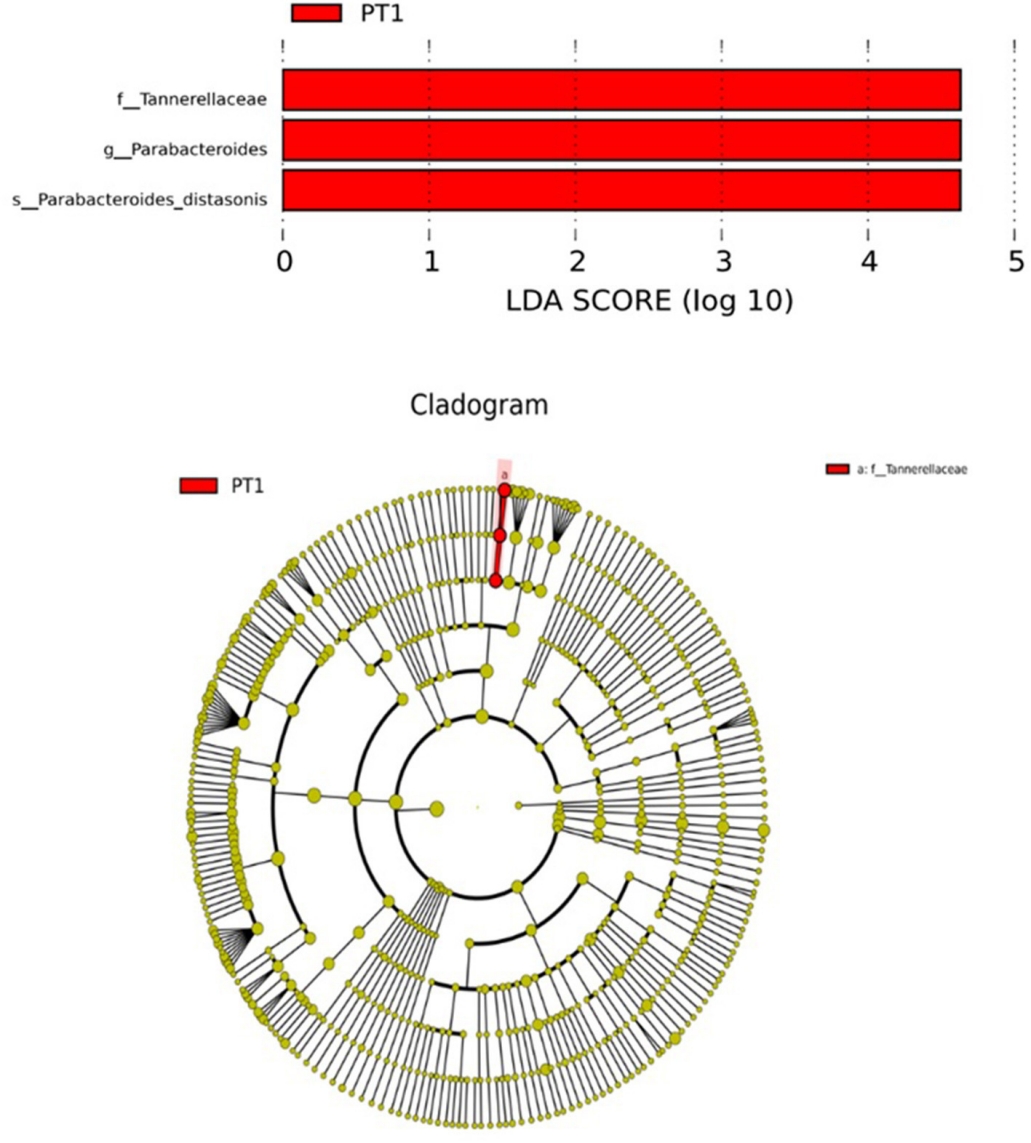
Differential intestinal flora between groups (LefSe).

Table 6 shows the alpha diversity of the cecal microbiota of the broilers at 42 days of age. There was no significant difference (*P* > 0.05) in the alpha diversity (including observed species, Shannon, Simpson, and Chao1 indices) of the cecal microbiota between the control and PT1 groups. Tables 7, 8 show the dominant flora with a relative abundance >2% at the family and genus levels. Table 7 shows that the dominant bacterial groups at the family level were Ruminococcaceae, Lachnospiraceae, Rikenelaceae, Tannerellaceae, Bacteroidaceae and Enterobacteriaceae. The average relative abundances were 35.22, 12.54, 9.58, 8.03, 4.41, and 2.47%, respectively. Among them, Tannerellaceae in the PT1 group was significantly higher than that in the control group (*P* = 0.048). Table 8 shows that the dominant bacteria at the genus level were *Alistipes, Parabacteria, Akkermansia* and *Bacteroides*. The average relative abundances were 9.58, 8.03, 4.68, and 4.41%, respectively. *Parabacteria* in the PT1 group was significantly higher than that in the control group (*P* = 0.039). In this experiment, linear discriminant analysis (LDA) was used to reduce dimensionality and to evaluate the impact of significantly different species. The LDA threshold was set to be >4. Table 8 shows that the dominant bacterium in the PT100 group was Parabacteroides, and its relative abundance was significantly higher than that in the control group (*P* = 0.039). LEfSe analysis (Figure 1) further identified the species with significant differences between the groups.

**Table 6:** Alpha diversity of cecal microbiota.

**Items**	**Control**	**PT1**	**SEM**	***P*-value**
Observed_species	716.00	660.33	60.116	0.979
Shannon	6.73	5.98	0.251	0.211
Simpson	0.97	0.93	0.018	0.073
Chao1	832.96	799.16	104.491	0.780

**Table 7:** The dominant flora at the Family level.

**Item**	**Control**	**PT1**	**SEM**	***P*-value**
Ruminococcaceae	39.74	30.70	4.591	0.079
Lachnospiraceae	14.15	10.93	1.637	0.081
Rikenellaceae	10.34	8.82	1.397	0.352
Tannerellaceae	5.15	10.91	2.174	0.048
Bacteroidaceae	4.89	3.93	1.540	0.519
Enterobacteriaceae	2.54	2.40	1.833	0.958

**Table 8:** The dominant flora at the genus level.

**Item**	**Control**	**PT1**	**SEM**	***P*-value**
*Alistipes*	10.34	8.82	1.397	0.311
*Parabacteroides*	5.15^b^	10.91^a^	2.174	0.039
*Faecalibacterium*	4.82	4.55	1.591	0.954
*Bacteroides*	4.89	3.93	1.539	0.625

*Means with different superscript letters within a row are significantly different (P < 0.05)*.

## Discussion

Growth performance is the most direct index to reflect the growth of broilers. Improving animal growth performance is the key to increasing economic benefits. The application and research of plant tannins in the production of monogastric animals are relatively limited. In the early stage, raw feed materials (sorghum) might be supplemented with a high dose or high content of plant tannins in the diet, resulting in negative effects such as decreases in feed intake, protein digestibility and production performance of monogastric animals. Therefore, this supplementation is regarded as an antinutritional factor (15, 16). However, several studies in recent years have shown that adding an appropriate amount of plant tannins to the diet can promote the growth performance, antioxidant function and immune performance of monogastric animals (17, 18). Starčević et al. (19) showed that adding an appropriate amount of plant tannins to the diet can improve the growth performance of broilers. Our results showed that the addition of an appropriate amount of *Plotytarya strohilacea Sieb. et Zuce* tannins in the diet tended to reduce the F/G, but it affected the ADFI of broilers from 1 to 21 days of age. This is not completely consistent with previous research results (19). It may be the bitter and astringent taste of tannin, which affects the palatability and reduces the feed intake of broilers in the early stage (20). However, combined with the results of ADG and F/G in the early stage, it was found that it had no negative impact on broilers. This indicates that the tannins may not inhibit digestive enzymes at multiple sites in the digestive tract and do not affect the digestion, absorption and utilization of nutrients in broilers. In our study, adding an appropriate amount of tannins to the diet had a certain beneficial effect on the growth performance of broilers.

Tannins can effectively remove oxygen free radicals in animals and can improve the activity of antioxidant enzymes in animals to reduce oxidative stress (21). CAT is one of the main antioxidant enzymes in animals. It can scavenge oxygen free radicals (ROS and NO) and protect the body from oxidative stress (22). GSH-Px can catalyze the decomposition of hydrogen peroxide *in vivo*. It is also one of the main antioxidant enzymes in the body and is conducive to the functional and structural integrity of the cell membrane. T-AOC is a comprehensive index reflecting the antioxidant system of the body. In our study, we found that tannins could significantly improve the serum CAT and T-AOC activities of broilers in a dose-dependent manner. In addition, the addition of vanillin tannin also showed a quadratic correlation with GSH-Px activity. GSH-Px activity was the highest when 100 mg/kg tannin was added, which is similar to previous studies (23). For example, studies have found that the addition of plant tannins is conducive to the enhancement of the antioxidant function of broilers to protect the excessive oxidation of lipids (23). López-Andrés et al. (24) found that adding tannins to the diet can improve the antioxidant function of serum. Hua et al. (25) showed that the addition of tannins can significantly improve the serum T-AOC of rabbit under heat stress and reduce the effect of heat stress on the body. Faraha et al. (13) added plant tannins to a broiler diet to reduce the content of MDA in muscle. Ye et al. (26) found that adding tannins to the diet tended to improve CAT activity in vole livers. In summary, under the test conditions, tannin has strong antioxidant capacity, and the antioxidant effect is the best when 100 mg/kg tannin is added.

The intestinal tract is the main organ for digestion and absorption of broilers. The length and weight of each intestinal segment reflect the growth and development degree of each intestinal segment, and the development degree can reflect the functions of digestion and absorption. In this experiment, the corrected length of intestinal weight and the intestinal index were used to evaluate intestinal growth and development. It has been found that adding an appropriate amount of tannins can protect and promote intestinal health by reducing oxidative stress, promoting the proliferation of beneficial bacteria and inhibiting the proliferation of harmful bacteria (27, 28). In our study, the results showed that the dietary addition of *Plotytarya strohilacea Sieb. et Zuce* tannins did not significantly affect the intestinal correction length or index of broilers, which was inconsistent with the results of previous studies (27, 28). This discrepancy may be caused by the different extraction sources of added tannins and the concentrations of the active components, and the specific mechanism needs to be further studied.

Intestinal villus height, crypt depth and V/C are important indices to measure intestinal digestion and absorption capacity. Liu et al. (29) found that adding chestnut tannins to the diet can significantly improve the villus height of the jejunum of broilers under heat stress, but the crypt depth showed no significant change. Brus et al. (28) found that the addition of low concentrations of tannins (0.05 and 0.1%) to the diet can stimulate the proliferation of intestinal epithelial cells and promote the intestinal development of broilers. The results of our experiment were similar with the results of study of Brus et al. (28). The addition of an appropriate amount of tannin to the diet could significantly increase the villus height of the jejunum of 42-day-old broilers, and there was a significant linear correlation between the villus height of the jejunum and tannin concentration. In addition, there was a significant linear correlation between the V/C of the ileum and the concentration of tannin. In agreement with the growth performance of this experiment, the ADFI of broilers decreased, but the ADG did not decrease. It is speculated that tannins can increase the area of nutrients absorbed by broilers' small intestine to reduce the negative impact of tannin's bitter taste. This may be because low-dose tannins can promote the proliferation of intestinal epithelial cells and protect DNA integrity in the process of cell proliferation (28) to promote the healthy development of the intestine and benefit production performance.

In this experiment, third-generation high-throughput sequencing technology was used. Third-generation full-length amplicon sequencing mainly amplified the full-length 16S sequence to construct the SMRT bell library. The community structure differences of different samples and groups were then obtained through analysis. According to the results of growth performance and antioxidant function, the differences in cecal microflora between the control and PT1 groups were further studied. It was found that the addition of 100 mg/kg tannins in the diet could significantly increase the relative abundance of *Parabacteroides*, which were also the dominant bacteria in broilers. Wang et al. (30) showed that *Parabacteroides* have anti-inflammatory, immune regulatory and metabolism effects, which are conducive to the health of the body. Kverka et al. (31) showed that *Parabacteroides* can significantly reduce the severity of intestinal inflammation in acute and chronic colitis model mice. Koh et al. (32) found that *Parabacteroides* can increase the expression of intestinal tight junction proteins, maintain the integrity of the intestinal barrier and be conducive to intestinal health. According to the results of growth performance, antioxidant and intestinal morphology, it is speculated that tannins can improve antioxidant function and promote the growth performance of broilers by increasing the abundance of *Parabacteroides*, maintaining the integrity of the intestinal barrier and improving blood biochemical indices.

## Conclusion

The addition of an appropriate amount of tannin in the diet can improve the serum antioxidant function, intestinal morphology, and growth performance, which may be related to the increase of the abundance of intestinal *Parabacteroides* by tannin. Taking into account growth performance, oxidation resistance and intestinal morphology, the recommended dosage of tannin in broiler is 100 mg/kg.

## Data Availability Statement

The data presented in the study are deposited in the figshare repository, https://doi.org/10.6084/m9.figshare.17078465.v1, accession number 17078465.

## Ethics Statement

The animal study was reviewed and approved by the Academic Committee of Southwest Forestry University.

## Author Contributions

ZT analyzed the data and drafted the manuscript. FL raised broilers and collected cecal samples. LL analyzed and interpreted the data. FW conceived and designed the manuscript. AG reviewed the manuscript and given critical suggestions and comments. All authors read and approved the final manuscript.

## Conflict of Interest

The authors declare that the research was conducted in the absence of any commercial or financial relationships that could be construed as a potential conflict of interest.

## Publisher's Note

All claims expressed in this article are solely those of the authors and do not necessarily represent those of their affiliated organizations, or those of the publisher, the editors and the reviewers. Any product that may be evaluated in this article, or claim that may be made by its manufacturer, is not guaranteed or endorsed by the publisher.
